# Book Reviews

**Published:** 1829-07

**Authors:** 


### CRITICAL ANALYSES.

Quæ laudanda forent, et quæ culpanda, vicissim

Illa, prius, cretâ; mox hæc, carbone, *notamus.*—Persius.

*Pathological and Practical Researches on Diseases of the Stomach, the Intestinal Canal, the Liver, and other Viscera of the Abdomen.* By John Abercrombie, m.d. Fellow of the Royal College of Physicians of Edinburgh, &c. and first Physician to his Majesty in Scotland.—8vo. pp. 396. Waugh and Innes, Edinburgh, 1828.

The ability with which Dr. Abercrombie has conducted his pathological investigations, gives him a place amongst the most useful writers of the present day. Two very desirable, yet not perhaps very common, features characterize his previous labours. He has shown himself to be free from the shackles of any exclusive system of pathology, and he allows diseases to retain their real characters, even should they be opposed to the abstract and artificial arrangements of nosology. Above all, he has been careful to form no general conclusions upon imperfect evidence; the besetting sin of so many medical writers. Although he has drawn very freely from various sources, in order more freely to illustrate the practical points he has discussed, we have not found him under the sway of that indiscriminate and injudicious passion for quotation which too often fills a volume, but adds nothing to the improvement of the reader of it. His practical experience ensures us judgment in his selections from the works of others.

As we have formed a favorable opinion of the previous investigations of Dr. Abercrombie, we naturally approach the present volume with the belief that we shall find in it much practical matter, that will deserve the studious consideration of medical practitioners. In all his researches, the object of Dr. A. has been to furnish facts in a concise and accessible form, and to advance to conclusions by the first step of the most cautious induction

The volume before us is divided into five parts, in reference to the five organs to which it relates, namely, the stomach, the intestinal canal, the liver, the spleen, and the pancreas. The two former are treated of at some length, with a view both to pathology and practice; and the three latter are considered with a more immediate reference to their pathological changes. The author first takes a general view of the various structures which enter into the formation of the stomach and intestinal canal, and of the principal morbid conditions to which they are subject. The peritoneum is liable to acute and chronic inflammation, and to various remarkable changes of structure, some of which result from inflammation, while others seem to have a different origin. The first effect of a certain low degree of inflammatory action upon serous membranes appears to be simply an increased deposition of the serous fluid; and in this manner it is probable that a certain state of these membranes, which, if not actually inflammatory, closely borders upon it, is sometimes relieved; the increased quantity of fluid being afterwards absorbed, and the parts thus recovering their healthy relations. In different states of the disease, remarkable varieties occur in the characters of the fluid which is deposited. In one case it is opaque and milky, or it contains shreds of floculent matter, or it has all the sensible properties of pus. All these varieties of the effused fluid are sometimes found without any remarkable change in the membrane itself, but in general it has undergone some considerable deviation from the healthy structure. The first is a slightly softened and thickened state of the membrane, giving it somewhat the appearance of a part which has been boiled. This Dr. A. thinks is commonly connected with the opaque milky deposition. The more common appearance consists in the surface being covered by a coating of false membrane. This may be connected with the milky floculent fluid, or with fluid which has all the sensible qualities of pus, or with an entirely limpid fluid.

"In the latter case, the deposition on the surface of the membrane will prevent the re-absorption of the fluid, so that the accumulation, which might otherwise have disappeared, will thus become a permanent dropsy of the cavity, provided the disease has not existed in such a form as to be speedily fatal. This state of parts is often seen most remarkably in the cavity of the pleura, the cavity being full of a limpid fluid, while it is lined by a complete and uniform cyst of false membrane. We are entirely unacquainted with the causes which regulate these varieties in the deposition from inflamed serous membranes. Under the influence of inflammation, also, whether acute or chronic, serous membranes are liable to form adhesions betwixt their opposite surfaces; and this may consist of simple adhesion with very little appearance of any interposed substance, or there may be an interposition of false membrane, which is often of very considerable thickness." (P. 4.)

In their structure serous membranes are liable chiefly to three morbid conditions: first, simple thickening, which is seen most strikingly in the peritoneum, which is sometimes found thickened in a most remarkable degree, and it appears to be the result of inflammation which has gone on in a chronic form. 2. Tubercular disease; the whole surface of the membrane being found studded with innumerable tubercles, generally of a small size and of a firm consistence. We saw a good example of this form of disease many years ago. The patient had not complained of any symptoms which indicated any affection of the peritoneum, but, upon dissection, the whole surface of the membrane was found thickly studded with small opaque bodies, which at first sight presented very much the appearance of small-pox pustules. Around the basis of these morbid growths there was no appearance of inflammation, nor was there any effusion into the cavity of the peritoneum. 3. There is another affection, often met with in the peritoneum, which appears distinct from tubercular disease. The membrane is covered by nodules of various shapes and sizes, of a semi-pellucid character and smooth rounded surface. The masses of this substance are sometimes of great size, and a large extent of the peritoneum may be found covered by them. This is the disease described by Dr. Baron, and supposed by him to be of the nature of hydatids. Dr. Abercrombie states that, although, on a first inspection, it has a resemblance to hydatids, in the specimens which he has had an opportunity of examining it appeared to be entirely of a different nature. "The nodules were of a uniform firm gelatinous consistence, or sometimes more dense at the centre than at the circumference. They did not appear to be covered by any cyst, and they were entirely soluble in boiling water."

The second structure is the muscular coat. We know little of the diseases of the muscular fibre, except in as far as relates to derangement of its functions. In a muscular covering which invests a cavity, the principal deviations from the healthy state appear to be the following: 1. A morbidly increased, but uniform and harmonious action; 2, a morbidly increased, but partial and irregular action; 3, diminution or loss of muscular power; 4, thickening of the muscular coat has also been described by some of the French writers, particularly as occurring in the stomach, constituting what they term hypertrophia, though some of them appear to apply this term to a general thickening of all the coats.

"Inflammation seems also to destroy the action of muscular fibre. Thus, intestine which has been highly inflamed is generally found in a state of great distention, showing the complete loss of its healthy muscular action; and, if the disease has gone on until the intestine has either become ruptured or has given way by ulceration, it is found to have fallen together like an empty bag, without any appearance of muscular contraction; whereas healthy intestine, when it is empty, contracts uniformly into a round cord. In regard to the immediate effects of inflammation upon muscular fibre, there is considerable obscurity; but one point may be considered as known and established, which is of considerable importance for our future inquiries, namely, that a result of inflammation in muscular fibre is gangrene. When, therefore, we find gangrene in the intestinal canal, we have reason in general to conclude that inflammation has existed in the muscular coat: for we shall afterwards find grounds for believing that it may exist in each of the coats separately without affecting the others, but giving rise to most important diversities in the symptoms." (P. 6.)

The third structure to which the researches of the author refer is the mucous membrane. The structure and functions of this membrane are briefly described. We have to attend to various forms of disease in mucous membranes connected with their peculiarities of structure. 1st. Inflammation and its consequences. From the lowest degree of inflammation on a mucous membrane appears to arise simply an increase of its proper secretion, more or less changed in its qualities from the healthy condition. In another state of inflammation we have aphthous crusts, and in a third the deposition of false membrane. "This last we see most remarkably in the bronchial membrane; it is also met with, though more rarely, in the mucous membrane of the intestines." We have at this moment a woman under our care, labouring under the disease denominated by Mason Good "diarrhœa tubularis." This patient has for some weeks discharged membrane-like tubes and flakes, which had been thought to be part of the mucous membrane of the intestines. The substance discharged in this case is considerable in quantity, and somewhat resembles the fibrous exudation thrown forth from the trachea in croup. In some instances this secretion has assumed the exact shape of the intestine.

In a more advanced stage, the mucous intestinal membrane may be softened from inflammation, or an ash-coloured pulpy degeneration of portions of the membrane may occur, which fall out and leave spaces, and perhaps pass into ulceration. A considerable extent of the membrane is occasionally found in a state of uniform dark softening, resembling gangrene. Adhesion of the opposite surfaces of the mucous membrane is sometimes met with, producing complete obliteration of the canal; but this Dr. A. states is very rare. The mucous membrane is apt to become thickened from long-continued chronic inflammation. In this manner is formed stricture of the urethra, and a similar diminution of the area of the intestinal canal.

Diseases of the follicles, or simple glands of the membrane, is a subject involved in much obscurity, but it seems to promise some interesting results. "The follicles appear to be liable to a vesicular or pustular disease, which passes into small, defined, distinct ulcers, quite unconnected with any disease of the mucous surface. Disease of a tubercular character is often met with on the mucous membranes, and in the cardia, pylorus, and extremity of the rectum: it often assumes a scirrhous character.

Having given a general sketch of the various morbid conditions to be considered in regard to the intestinal canal, the author enters upon the "*Pathology of the Stomach.*"

The great proportion of affections of this important organ appear to be entirely of a functional nature, leaving no morbid appearance that can be discovered after death. In others the appearances are too doubtful to lead to any precise principle in pathology. "In a practical point of view, also, this is perhaps more encumbered with uncertainty than almost any other department of medical practice; for the diseases are so much under the influence of moral and other adventitious causes, that the action of remedies is aided, modified, or counteracted, in a manner which entirely eludes our observation, and is often altogether beyond our control." Dr. Abercrombie considers affections of the stomach under three classes:

"1. Affections of an inflammatory kind, including ulceration and its consequences.

"2. Affections which more properly come under the class of organic.

"3. Functional affections, embracing a slight outline of the subject of dyspepsia.

*Of the inflammatory affections of the stomach, and ulceration.*—The author observes, that although acute gastritis is described by all systematic writers, it is very difficult, in the records of pathology, to find a pure example of it in an idiopathic form. He has often been astonished to find how seldom the stomach shows marks of inflammation, even when the organs most nearly connected with it have been inflamed in the highest degree. A case of pure inflammation of the peritoneal coat of the stomach Dr. A. has never seen; neither does he find it described by any writer. The disease which we call gastritis is to be considered as seated chiefly or entirely in the mucous membrane, and even here it is very rare as an acute and idiopathic disease. The symptoms which are usually described as those of gastritis, are pain and tenderness in the region of the stomach, urgent vomiting and fever; but, from the facts upon which we can rely, it does not appear that the symptoms are so uniform as systematic writers would lead us to believe. On the other hand, De Haen, Stohl, and Frank, describe cases of what they term inflammation and gangrene of the stomach, in which none of the usual symptoms of gastritis had occurred; and other cases which had exhibited all the symptoms of gastritis, while no appearance of inflammation could be discovered on dissection. It is also very certain that symptoms closely resembling those ascribed to gastritis frequently subside under treatment the very reverse of that which would have been applicable to inflammation.

"To these circumstances we have to add the important facts ascertained by Dr. Yelloly and others. In numerous cases of persons who died of other diseases, without any symptoms in the stomach, and in the bodies of criminals who had been executed, they have pointed out appearances which might have been considered as distinctly indicating inflammation of the mucous membrane of the stomach." (P. 15.)

Dr. A. presumes it will now be admitted that the term inflammation ought not to be applied to any appearances consisting of mere change of colour or increased vascularity, without some decided change in the structure of the part, or some of the actual results of inflammation.

"Upon the whole view of the subject, the conclusion seems to be, that we are still very much in the dark in regard to idiopathic acute gastritis. For my own part, I have never seen a case which I would consider as being of this nature; and I am disposed to regard as points not yet ascertained, what are the characters exhibited by the mucous membrane of the stomach in the earlier periods of acute gastritis, and in what they differ from appearances which may exist without any symptoms of gastric disease, or take place after death. If we might proceed in any degree upon the analogy of the corresponding affection in the mucous membrane of the bowels, I should be inclined to suppose that the disease exists under two forms: that in the one it is seated chiefly in the follicles or simple glands, in the other in the mucous membrane itself; that, in the former case, it would consist, in its early stage, of detached and minute pustules or vesicles, and would terminate at an early period in minute and detached ulcers; and that, in the other, it would exhibit, in its first stage, the appearance of defined portions of the mucous membrane, of a red or livid brown colour, and sensibly elevated above the level of the surrounding parts; these portions afterwards terminating by softening or ulceration, or passing into a chronic state of disease with ulceration, thickening, or fungoid elevations upon the diseased parts. This is in some measure conjectural; but I think we may safely assert that, in this investigation, nothing can be founded upon a mere general or extensive redness of the membrane, discoloration, or increased vascularity, whether more or less extensive, venous turgescence, extravasation of blood into the cellular texture, or upon any appearance which consists of mere change of colour, without any decided change in the structure of the part." (P. 15.)

Leaving this part of the subject for further investigation, the author proceeds to another of much practical importance, in reference to which we have many interesting facts on which we can proceed with confidence. We have every reason to believe that the mucous membrane of the stomach is liable to inflammation in a chronic form, which often advances so slowly and insidiously that its dangerous nature may be overlooked, until it has passed into ulceration, or has even assumed the characters of organic and hopeless disease.

"Further, we shall find that even ulceration may exist in the stomach without producing any symptoms of an alarming nature, until it gives rise to an attack which is very speedily fatal. In the early stages of this affection, the prominent symptoms are often such as merely indicate derangement of the functions of the stomach, and are apt to be included under the general term dyspepsia. The patient perhaps complains of extreme acidity, eructations, flatulence, and oppression of the stomach after eating. There is generally some degree of pain in the region of the stomach, but it varies very much both in its degree and its duration. In many cases it is complained of only after eating, continues in considerable severity while the process of digestion is going on, and subsides when that process is completed. The appetite is often unimpaired, but the patient is afraid of taking food on account of the uneasiness which is produced by it, and he is entirely free from complaint when the stomach is empty." (P. 17.)

Vomiting is apt to occur, but in the early stages is only occasional, and is ascribed to some error in diet, or other accidental cause. Afterwards it becomes more frequent, but still without that regularity which would seem to indicate serious disease. By attention to diet it may be in a great measure prevented, and in this manner the disease may go on for months without exciting alarm. The vomiting then, perhaps, becomes more frequent, and the uneasiness in the stomach more permanent, until the patient either sinks by gradual wasting, or is suddenly cut off by one of those rapid attacks which Dr. A. afterwards particularly describes. In all the forms of this insidious disease there is great diversity in the symptoms. In many cases it will be found that little or no uneasiness had ever been complained of until the attack takes place, which is fatal in a few hours. "An important circumstance, therefore, in the history of this affection is, that it may run its course almost to the last period without vomiting, and with scarcely any symptom except the uneasiness which is produced by eating, and which subsides entirely in a few hours after a meal." The author gives a case in illustration of this fact. In some cases the prominent symptom is a very copious discharge from the stomach of a clear glairy fluid, like the white of eggs. In a woman, mentioned by Andral, this discharge amounted to about four pints in twenty-four hours, and she never vomited either food or drink. This discharge is sometimes streaked with a black matter, or is entirely of the colour of chocolate, and not unfrequently is mixed with grumous blood.

From this chronic inflammation of the mucous membrane results very frequently ulceration, in various forms, of the inner surface of the stomach. There may be one or more small defined ulcers of limited extent, with evident loss of substance and rounded and elevated edges, every other part of the stomach being in the most healthy state. The ulcer may be confined entirely to the mucous membrane, or all the membranes may be perforated. An interesting case of this latter form of disease is given in our Journal for April 1825, p. 289, by Mr. Griffiths. In this instance the perforation of the stomach is described as "looking much as if it had been made with a punch." Precisely the same description is also given of similar cases related by Dr. Ebermaier,* who remarks that the real nature of the malady was in no instance suspected by the physicians. Death sometimes occurred unexpectedly, almost in the midst of apparent health. Dr. E. is of opinion that these perforations do not result from ordinary chronic inflammation. Similar ulcerations, perhaps the size of a shilling, but complicated with thickening and induration of the parietes of the stomach around the ulcer, are sometimes met

* London Med. and Phys. Journal, October 1828, p. 302, and November 1828, p. 422.—An excellent article upon this subject, which still requires elucidation, is contained in the 46th volume of the Dict. des Sc. Medicales, p. 314, art. *Perforation*, with plates.—Rev.

with. In the progress and terminations of this disease there is considerable difference.

"A singular variety in the appearances is to be referred to before leaving this part of the subject. Though a complete perforation of the stomach by ulceration may have taken place, it is frequently found that an adhesion had been formed to some of the neighbouring parts, most commonly the liver, in such a manner that a portion of the surface of the liver supplies the place of the portion of the stomach that has been destroyed, and thus no escape of the contents takes place." (P. 21.)

This remarkable circumstance Dr. Abercrombie exemplifies by a case, which was afterwards fatal by a small perforation immediately adjoining the portion where this adhesion had been formed.

"Another important modification arises from adhesion of the stomach to the arch of the colon, and a communication being formed between them by ulceration." An instance of this kind is also given by the author, and several cases illustrative of the principal modifications of ulcerations of the stomach, and the insidious manner in which such diseases are apt to advance, with symptoms which are liable to be considered merely dyspeptic. The first case is especially interesting. The disease appeared to be seated entirely in the mucous follicles. One fact particularly worthy of notice was "the activity of the symptoms in the early stages, probably while the follicles were in a state of inflammation, and the obscurity of them when the disease was more advanced; likewise the proofs that many of the follicles had been in a state of ulceration, and had cicatrised; while, at the time of the patient's death, not above two or three were in a state of ulceration.

It must be evident, from the description which has been given of the uncertain and various symptoms which attend both the commencement and progress of the different species of the above-named affections of the stomach, that much difficulty must attend the diagnosis. Dr. A., from the facts he has stated, thinks there is every reason to conclude that the dangerous affection referred to in the preceding observations exists in two conditions:

"Namely, chronic inflammation of a defined portion of the mucous membrane of the stomach, or the mucous follicles; and the termination of this by ulceration. In both these conditions it may probably be the subject of medical treatment; for we have reason to believe that the inflammation may be arrested and prevented from passing into ulceration, and that the ulceration may heal before it has become connected with any permanent change in the organization of the part. Hence appears the importance of minutely watching the progress of the disease in its early stages, in which only it is likely to be treated with success. The difficulty here is in the diagnosis; the disease often assuming the character of a mere dyspeptic affection through a great part of its progress, while in fact a morbid condition of a very serious nature is going on, which would require treatment in many respects very different from that adapted to dyspepsia." (P. 46.)

"Amid such a diversity of symptoms as occur in connexion with this disease, our chief reliance in the diagnosis must probably be on a careful examination of the region of the stomach itself, with the view of discovering the existence of tenderness referred to a particular part. This examination should be made with the most minute attention at various times, both when the stomach is full and when it is empty. If induration be discovered, the character of the case will be obvious; but we have seen that most extensive ulceration may exist without any induration, and likewise that extensive induration may exist without being discovered by external examination.

"Other important cautions in regard to the diagnosis will be learned from the cases which have been described. In particular, we should not be deceived either by the pain having remarkable remissions and the patient enjoying long intervals of perfect health, or by remarkable alleviation of the symptoms taking place under a careful regulation of diet; for these circumstances we have found occurring in a very striking manner, while the disease was making progress to its fatal termination." (P. 48.)

*Treatment.*—When the disease is detected at an early period, our treatment must consist chiefly of free topical bleeding, blistering, issues, or tartar-emetic ointment. Food must be small in quantity and mild in quality, as farinaceous articles and milk. The stomach should not be distended even by the mildest articles. Bodily exertion is improper, "and hence the importance of endeavouring to distinguish the disease from mere dyspepsia, as the regimen and exercise which are proper and necessary in dyspepsia would in this case be highly dangerous." In the early periods medicine can do little more than regulate the bowels. In the more advanced stages, when ulceration may be suspected, the same remark will apply to external applications and regimen.

"Benefit may now be obtained by some internal remedies, such as the oxide of bismuth, lime-water, and nitric acid; and in some cases small quantities of mercury appear to be useful. Small opiates, combined with articles of a mucilaginous nature, appear frequently to be beneficial; likewise articles of an astringent nature, such as kino, alum, and the rhatany root. The arsenical solution has also been recommended, and small doses of the nitrate of silver; and, in several instances in which I suspected this disease to be going on, I have found remarkable benefit from the sulphate of iron. Whether the disease can be cured after it has advanced to ulceration, must indeed remain in some degree a matter of doubt; because, in a case which has terminated favorably, we have no means of ascertaining with certainty that ulceration had existed. In some of the cases, however, which have been described, we have seen every reason to believe that some of the ulcers had cicatrised, though the disease had afterwards gone on to a fatal termination; and, from what we observe in the intestinal canal, we can have little doubt that simple ulceration of the mucous membrane may cicatrise. I am satisfied that I have seen the cicatrices of such ulcers when the patient has died of another disease, after having been for a considerable time free from any symptom in the bowels." (P. 49.)

Under the use of strict diet, the worst cases have sometimes recovered. The following is an example:

"A female, whose age is not mentioned, had for a considerable time laboured under symptoms which were supposed to indicate scirrhus of the pylorus, and her case had been regarded as entirely hopeless. She suffered severe pain in the stomach when the smallest quantity of food was taken in, with great tenderness upon pressure and constant vomiting, which occurred regularly about the same period after eating at which it usually takes place in affections of the pylorus. A variety of treatment had been employed without benefit, when Dr. Barlow determined upon trusting entirely to regimen, by restricting her to a diet consisting wholly of fresh-made uncompressed curd, of which she was to take but a tablespoonful at a time, and to repeat it as often as she found it advisable. On this article she subsisted for several months, and recovered perfect health." (P. 51.)

Dr. A. has seen some cases of that species of aphthous affection of the mouth, fauces, and larynx, to which the French apply the name of Diphtherite. It is an epidemic chiefly affecting children, and frequently fatal. A description of the symptoms and treatment of this disease is given.

In the next section the author treats of *organic diseases of the stomach.* Under this head are briefly mentioned, induration and thickening of the coats of the stomach, diseases of the pylorus, and disease of the cardia.

*Pathology of Dyspepsia.*—All we know of the process of digestion is, that it is the result of the combined action of the gastric juice, and of a peculiar muscular motion of the stomach. In healthy digestion it appears that no gas is generated in the stomach, but that a certain quantity is evolved in the farther progress of the alimentary matters through the intestines, especially in the colon; and it is said to be composed of carbonic acid, hydrogen, and azote, in various proportions.

"When these actions are in any degree deranged or deficient, the alimentary matters are not converted in the regular manner into healthy chyme; but, remaining perhaps longer in the stomach than in the healthy state of the process they would do, they undergo, in a greater or less degree, those chemical changes which would happen to them in other circumstances. Hence the generation of acidity, the evolution of gases of various kinds, and the lodgment in the stomach of matters imperfectly digested, partly fermented, perhaps partly putrid: hence, also, irregular muscular contractions, arising from the morbid stimuli thus produced, giving rise to regurgitations of matter into the œsophagus, eructations, and perhaps vomiting; or, the muscular coat yielding to the distending force of the evolved gaseous fluids, there are produced painful distention, oppression, and anxiety, or, in other words, a paroxysm of dyspepsia." (P. 68.)

Dr. Abercrombie admits that the dependence of the function of digestion upon the influence of the eighth pair of nerves, is among the most beautiful discoveries of modern physiology, but he is not aware that any practical results have hitherto been deduced from it.

In the present section the attention of the reader is directed to those cases in which derangement of the stomach is of a functional nature, or not connected with any change of structure, either of the stomach itself or of any of the neighbouring parts. It is extremely difficult to ascertain the exact nature of these functional derangements, as they are merely impaired actions of living parts. The muscular action of the stomach may be deficient, so that the alimentary matters remain in it too long, are imperfectly changed, and pass into chemical decompositions.

"We know the state of the urinary bladder, in which its muscular action is lost or very much impaired, and in consequence of which it is gradually distended, so as to hold an enormous quantity of fluid; and, when emptied by the catheter, it does not contract equally, as in the healthy state, but falls flat like an empty bag. A state analogous to this we not unfrequently see in the stomach on dissection; a state in which it appears much enlarged, and collapsed by flattening, without healthy contraction." (P. 69.)

There may be a deficiency of the corresponding and harmonious intestinal action, interfering with the second stage of digestion, and giving rise to imperfect chylification and various morbid actions in the upper intestines. The various fluids concerned in the process of digestion may be deficient in quantity or morbid in quality. The mucous membrane of the stomach may be morbidly irritable, and the muscular coat be too easily excited to action.

"If this occur in the stomach, the articles of food will not be allowed to remain in it a sufficient time for healthy digestion; but, after producing much uneasiness, they will either be rejected by vomiting or propelled in a half-digested state into the intestine, there to prove a source of new irritation. This is probably the state to be afterwards more particularly referred to, in which animal food produces much uneasiness in the stomach, often followed by vomiting; but in which digestion goes on in a healthy manner, on a regimen restricted to farinaceous articles and milk. If the irritability occur in the intestine, the articles may undergo their proper change in the stomach, but will be propelled too rapidly through the intestinal canal, without time being afforded for the complete process of healthy chylification; and accordingly, in many affections of the stomach and bowels, we see articles, even of the most digestible kind, pass through partially digested, or sometimes entirely unchanged." (P. 70.)

The author has no intention of entering fully into the treatment of indigestion. The following rules he considers important.

"1. It appears that the muscular action of the stomach is both more vigorous and more extensive when its contents are in small quantity, than when it is much distended; and, if we suppose the fluids of the stomach to be secreted in nearly a uniform quantity, their action must also be greatly regulated by the quantity of matter which they have to act upon: hence the indispensable importance, in dyspeptic cases, of restricting the food to such a quantity as the stomach shall be found capable of digesting in a healthy manner. This is unquestionably the first and great principle in the treatment of indigestion; and, without invariable attention to it, no other means will be of the smallest avail.

"2. It appears that various articles of food are of various degrees of solubility in the stomach. When, therefore, digestion is apt to be easily impaired, it will be of the greatest importance not only to avoid articles which are of difficult solution, but also to avoid mixing various articles which are of different degrees of solubility. Attention to this rule will probably favor, in a great measure, the process of chymification going on in a regular and healthy manner, by avoiding a state in which the solution of one article may be more advanced than that of another. The articles of most easy solution appear to be solid animal food and white fish, both plainly dressed; vegetables are less soluble; and, among the articles of more difficult solution, appear to be fatty substances, tendinous and cartilaginous parts, concrete albumen, the epidermis of fruits, and, according to some, mucilaginous and sweet vegetables. From some experiments of Sir Astley Cooper, it is supposed that the solubility of animal food is in the order of pork, mutton, veal, beef. Articles in small pieces are much more speedily dissolved than in larger, the action being found to begin at the circumference of the portion; and hence the importance of careful mastication.

"3. If digestion go on more slowly and more imperfectly than in the healthy state, another important rule will be, not to take in additional food until full time has been given for the solution of the former. If the healthy period be four or five hours, the dyspeptic should probably allow six or seven. The injurious infringement of this rule by a breakfast, a meat lunch, and a dinner, all within the space of seven or eight hours, is too obvious to require a single observation." (P. 71.)*

Dr. Abercrombie despairs of offering much novelty of remark respecting the medical treatment of dyspeptic complaints. One caution, however, has always appeared to him of the utmost importance, and we know that it is too little attended to, however frequently it may have been enforced. It consists in regulating the bowels, which in some complaints are disposed to be inactive, by the daily use of very small doses of laxatives combined with tonics, so as, without ever purging, to imitate at all times that moderate but regular action which constitutes the most healthy state of the bowels. It must, however, be admitted that an attack of dyspepsia may arise from an overloaded state of the bowels, which may be so obstinately constipated as to demand the exhibition of powerful purgatives; but the practitioner should certainly confine himself as much as possible, in dyspeptic complaints, to the employment of mild laxatives.

The author apprehends, and very justly too, that no small injury is done by the indiscriminate use of mercury. Calomel is the catholicon of the present day, and we fear it is not more empirically employed by the public than the profession. Dr. Abercrombie is of opinion that, in all disorders of the stomach, mercury, in any form, or in any quantity, ought not to be employed when the desired effect can be accomplished by any other means. If, however, the affection of the stomach is connected with hepatic derangement, a cautious use of mercury is recommended.

* Dr. James Johnson, in his Essay on the Morbid Irritability of the Stomach, has very clearly pointed out the necessity and advantage of strict attention both to the quantity as well as the quality of food taken by dyspeptic patients.—Rev.

Some good practical observations are made upon the different forms of gastrodynia.

" It is difficult to say what remedies are best adapted to each of these forms of gastrodynia. I have found nothing of more general utility than the sulphate of iron, in doses of two grains, combined with one grain of aloes and five grains of aromatic powder, taken three times a day. Oxide of bismuth combined with rhubarb in the same manner, is also frequently very useful; likewise lime-water, and small opiates. When the affection proves more obstinate, it must be treated by topical bleeding and blistering, with farinaceous diet." (P. 77.)

Sympathetic affections of the heart are among the most troublesome symptoms that accompany affections of the stomach, and are always alarming to the patient, and not unfrequently a source of much perplexity and error to the practitioner.

" They appear under various forms, and frequently assume, in a very great degree, all the characters of fixed disease of the heart or large vessels. The slightest and perhaps the most common form consists of a momentary feeling of a rolling or tumbling motion of the heart, like that which is produced by a sudden surprise or fright, and it is accompanied by an intermission of the pulse. This feeling may be repeated only once or twice at a time, and occur at long intervals; or it may return in rapid succession, for half an hour or an hour together; or it may be felt occasionally, at irregular intervals, for several days or weeks, or for a still longer period. It is sometimes accompanied by a feeling as if the heart were violently grasped. In other cases, the affection assumes the form of continued fits of palpitation, or strong and irregular action of the heart, which continue without any remission for an hour or more at a time, and recur in this manner daily, or several times in a day, for a length of time; or recur at uncertain intervals. In other cases, again, these fits of palpitation continue for several days together. They are, of course, accompanied by irregularity of the pulse, when the action of the heart is itself irregular; but frequently there is no irregularity in the action, the affection merely consisting of a strong pulsation, which the patient feels or hears throbbing in his ear, and can count distinctly by the sound, especially when he lies in bed. In other cases, again, there is only an increased frequency of the action of the heart, showing itself by paroxysms of quick pulse, accompanied with a feeling of anxiety, continuing for an hour or two at a time, without any irregularity." (P. 81.)

The various forms of this affection are principally distinguished from disease of the heart by the regularity of the pulse, and the natural action of the heart during the intervals between the attacks, and also by the symptoms being obviously connected with disorders of the stomach, and by their being relieved by treatment directed to that organ; and "particularly by the symptoms being most apt to occur while the patient is at rest, especially after meals, not being increased by bodily exercise, but rather relieved by it, and not being excited by such bodily exertion as we should naturally expect immediately to influence a disease of the heart."

Several cases are detailed, in which the function of the heart was seriously disturbed by gastric derangement.

In concluding this part of the subject, Dr. A. takes occasion to deprecate the vague and undefined use of the term "dyspepsia" which some writers have sanctioned. He observes, "when we find these writers talking of a stage of dyspepsia in which it terminates by ulceration, or various organic affections of the parts concerned, I cannot avoid considering them as using a phraseology which is at variance with the principles of sound investigation, and calculated to obscure a subject of the utmost practical importance." In this opinion we perfectly agree, although we are aware of the ability with which a different view of the subject has been advocated.

In an Appendix to the Pathology of the Stomach, some observations are made, 1st, on derangement of the stomach by tumors attached to it externally, without disease of its coats; 2d, outline of the pathology of the œsophagus; 3d, outline of the pathology of the duodenum. The following case, in illustration of the first kind of disease, is worthy of notice:

"A lady, aged about seventy, had been affected for more than thirty years with periodical vomiting, which occurred so regularly a few hours after meals, that, during the whole of this period, she had vomited a part of almost every meal. It was brought up without nausea or any unpleasant effort, and the affection had never injured her general health. I was in the habit of seeing her for several years, during which time she continued to enjoy good health, till she began to fall off rather suddenly, and died, after a short illness, with diarrhœa and rapid failure of strength.

"*Inspection.*—The only morbid appearance that could be discovered was a tumor, the size of a hazel nut or very small walnut, and resembling an enlarged gland. It lay in contact with the outside of the stomach, near the pylorus, and slightly attached to its outer coat, but without any appearance of disease in the stomach itself." (P. 90.)

A similar case is mentioned by MORGAGNI, in which the symptoms had gone on for twenty-four years: the only appearance was a slight induration of the pancreas.

We have now given a full abstract of the first division of this volume. At a future opportunity we shall present our readers with an account of the remaining subjects which the author has discussed. It would have been impossible to do justice to a work of so much practical merit in the compass of a single article.

---

*An Account of some of the most important Diseases peculiar to Women.* By Robert Gooch, M.D.—8vo. pp. 432. Murray, London, 1829.

There was a time, half a century ago, when it was common to find, in medical men who had risen to high eminence, not only great professional acquirements, but also distinguished literary and general scientific attainments, a combination of knowledge which was not less useful to the public than it was honourable and adorning to the individual and the profession at large. This race of highly polished physicians has now nearly passed away, and given place to a class of men in general more studious of being celebrated for their practical knowledge alone, than of enriching and embellishing their minds by an acquaintance with all the polite arts and sciences. Some, however, still exist, *rari nantes in gurgite vasto,* to save the honour of their times; and of these the author, whose book is now before us, affords an eminent example. To great professional and practical acquirements, he unites an intimate knowledge of polite literature, and of the arts and sciences in general.

Dr. Gooch has long been known to the public and the profession as a physician whose opinion, particularly in female complaints, was of great value; and his method of investigating disease has generally been supposed to bear a great resemblance to that pursued by the late Dr. Baillie. Possessing, then, a mind so highly endowed and so rare at the present day, it is not to be wondered at that the work ushered forth under the sanction of his name should have been received with respect, and perused with interest. High as Dr. Gooch's character has hitherto deservedly stood, it will receive, we will venture to say, no tarnish from the present volume, which, whether we regard it in a literary or medical point of view, must be considered to bear the stamp of superiority. The dedication and preface, looked upon merely as specimens of composition, are written in a very elegant and classical style, and cannot fail to attract the attention of the reader; while the interior of the volume displays a rich collection of facts and observations in reference to the diseases of which it treats, deduced from opportunities which few have enjoyed, and still fewer known how to employ.

After an instructive, although certainly rather an unusual preface, inasmuch as it is principally occupied by an address to students on the best manner of acquiring correct medical knowledge, the author proceeds to the consideration of *the peritoneal fevers of lying-in women;* diseases, on the real nature of which, some of the most distinguished cultivators of medical science have come to very opposite conclusions. This interesting subject seems to have engaged the attention of the author from the outset of his career, and has accordingly, from the long application of his talents, received much elucidation. It need hardly be stated that some writers, deservedly of great authority, have considered peritoneal or puerperal fever to be entirely of that character where bloodletting and evacuants could only be employed at the hazard of life, and where opium, wine, and other stimulants, were the only means capable of combating its alarming and rapidly fatal course; while, on the other hand, men of great celebrity have affirmed that the disease was an acute inflammation of the peritoneum, and that the proper method of treating it was by an active employment of the antiphlogistic regimen. Of the opinions of the supporters of these two very opposite doctrines, the author has given a very candid review, and has pointed out the probable grounds of the great discrepancy in their statements. The results of his own experience follow.

Not long after Dr. Gooch was appointed physician to the Westminster Lying-in Institution, he remarked that the peritoneal fever prevailed, or was epidemic, at particular seasons. The following is the description of the disease which fell under his notice at that time:

"The cases which were so numerous in these unhealthy seasons had the common symptoms and course of puerperal fever. They began a few days after delivery; the leading symptoms were diffused pain and tenderness, with some swelling of the abdomen, a quick pulse, which was generally at first full and vibrating. Sometimes it was small, but still it was hard and incompressible; The skin was hot, though not so hot as in other fevers; the tongue was white and moist; the milk was suppressed. As the disease advanced, the belly became less painful, but more swelled, and the breathing short; towards the end, the pulse was very frequent and tremulous, and the skin covered with a clammy sweat: even in this state the tongue continued moist and the mind clear, and death took place generally about the fifth day. On opening the abdomen, which was often as large as before delivery, the intestines were found distended with air, the peritoneum was red in various parts, its surface was covered with a coat of lymph; the intestines adhered to one another, and the omentum to the intestines; coagulable lymph was deposited on various surfaces, especially in the depressions between the convolutions of the bowels and on the omentum, on both which parts it often lay in large masses. The cavity of the peritoneum contained several pints of a turbid fluid, apparently serum mixed with lymph." (P. 41.)

This complaint at the commencement bore all the marks of acute inflammation, but in the course of two or three days it had much altered its character: the abdomen became less painful and tympanitic, the strength of the patient sank in an alarming degree, the pulse varied from 140 to 160; and, when the disease had reached this stage, the patient invariably became its victim.

In the first stage of the disorder, the treatment of the author was as follows:

"I now found that, provided I saw the patient within a few hours of the attack, I could generally arrest the disease. The mode of treatment was as follows: A vein was opened in the arm, with a wide orifice, so that the blood flowed in a full stream, and it was allowed to flow till the patient felt faint; the arm was then tied up, and her head was raised so as to encourage the faintness for many minutes. As soon as the faintness had subsided, she took from ten to twenty grains of calomel in a teaspoonful of arrowroot, and afterwards half an ounce of sulphate of magnesia, dissolved in beef-tea or thin gruel, every other hour, until several copious evacuations were procured from the bowels. When the patient had thoroughly recovered from her faintness, from ten to twenty leeches were applied to the painful and tender parts of the abdomen; when the leeches had fallen off, a bag, long and broad enough to cover the whole abdomen, was stuffed with hot poultice, which was spread so as to form a cushion nearly an inch thick: this was laid hot over the whole abdomen, and renewed so often as to keep up heat and moisture." (P. 46.)

Bleeding was afterwards repeated as long as the pulse remained firm and the abdomen painful; or local blood-letting, by means of leeches, according to the severity of the symptoms, was employed. "The active treatment," says Dr. Gooch, "that which will determine the fate of the patient, should be begun and ended during the first day: when employed later, it is under great disadvantages, and with many diminished chances of success."

The conclusion to which Dr. G. came as to the nature of the puerperal fever which prevailed occasionally between the years 1812 and 1820, was that it was "a fever attended by acute inflammation of the peritoneum; that the inflammatory stage was often very short, soon terminating in great and irremediable effusion into the peritoneum; that the disease was curable only in the inflammatory stage by active bleeding and purging; and that, although it was impossible to draw the line, and say when the inflammatory stage terminated in that of effusion, because it differed in length in different cases, yet that it was often incredibly short, and that the treatment had not a fair chance of success unless begun during the early hours of the disease. Thus my experience agreed in all the principal points with that which had been so forcibly stated to the public by Dr. Armstrong and Mr. Hey." (P. 62.)

The author soon, however, found that if a woman, after lying-in, had pain and tenderness of the abdomen, with a rapid pulse, it was *not* necessarily a case of peritoneal fever, for which the only remedy was early and active depletion, and that there were cases of lying-in women affected with pain and tenderness of the abdomen, which it is impossible to distinguish from the symptoms of real inflammation; with rapid pulse, and all the usual symptoms of peritoneal fever, which were not cases of that disease. They were rendered worse by an early and active depletion; nor, on dissection, did the body exhibit any marks of peritoneal inflammation. The author has related several cases of this disease, with all the symptoms of puerperal fever, which during one epidemic was generally fatal, and where no morbid appearance of the peritoneum could be discovered after death. The disease was relieved, and frequently cured, by means of opiates, and remedies of a similar nature. The author concludes this part of the subject by stating, "the experience of the last few years has brought me to this conclusion: that the sanguine hopes which were entertained a few years ago, that the peritoneal fevers of lying-in women are always of an acute inflammatory type, and are always to be cured by early bleeding and purging, as they were not borne out by the reasoning employed, so they have not been confirmed by subsequent experience." (P. 97.)

Such is precisely the result of our own observation, and we confess we are yet unable to distinguish with certainty those cases which may, from the commencement, be trusted to the *free* exhibition of opiates, from those which demand active and repeated bloodletting. In one case the pertinacious opposition of a patient afforded us a useful lesson. It was determined in consultation that she had peritoneal inflammation, and that venesection was to be promptly and freely had recourse to. To this practice she positively refused to submit. Leeches and fomentations were applied to the abdomen, and the Liq. Opii sedat. given in full doses. The pain of the abdomen, and its tenderness on pressure, soon subsided, and the patient speedily recovered.

In the treatment of two diseases so diametrically opposite, and yet distinguishable from each other with so much difficulty, the author very naturally asks, how are we to proceed? and, for the purpose of directing our conduct in this very essential particular, he has laid down some very judicious rules. The first and most important object is to apply the remedies, whatever they may be, at the very outset of the disease. Secondly, to approach the patient with a firm belief of the difficulty of diagnosis in these cases; and, lastly, practitioners of midwifery are directed to make themselves acquainted with those modes of treatment which appear to have been the most satisfactory. "The remedies for the efficacy of which there is most evidence are, first, bleeding and purging; second, emetic doses of ipecacuanha; third, opiates internally, and poultices externally to the abdomen; fourth, mercury given so as to affect the constitution; fifth, oil of turpentine." (P. 101.)

An attention to the nature of the prevailing epidemic is also of the greatest use in guiding the practitioner to the proper method of conducting the treatment of this hazardous complaint.

Such is an abstract of the observations of Dr. Gooch on this perplexing subject. With the candour which invariably accompanies real ability, he confesses that some parts of the subject are beset with difficulties; there is no assumption of knowledge which is yet to be obtained. Were we to object to any part of his remarks, it would be his calling that disease peritoneal fever in which he affirms no affection of the peritoneum exists. We think, also, he should have pointed out the great similarity between his second fever and that arising from inflammation of the veins of the uterus, which is generally mistaken for, and denominated, puerperal fever.*

Having entered so fully into this part of the work, we

* This species of fever has been fully and accurately described by M. Dance, Dr. Robert Lee, and other writers. The interesting practical paper of the last-named gentleman will doubtless be published in the next volume of the Transactions of the Med.-Chir. Society.—Rev.

find we have scarcely room to give more than a mere notice of the other diseases which have engaged the attention of the author.

Dr. Gooch's opinions on *puerperal insanity* will be perused with much instruction. He has shown, first, that in general the disease arises in constitutions enfeebled by nursing, or some previous debilitating cause, such as the depressing affections of the mind, than which no external agent can act more forcibly; secondly, that the disease is invariably rendered worse by bloodletting, and that the cure is best conducted under the soothing and "sustaining influence of opium:" deductions which no one, after attending to the reasoning of our author, will be inclined to dispute. To this part of the work are added some interesting thoughts on insanity as an object of moral science.

Of the *symptoms of pregnancy, and the diseases resembling it*, the reader will find an excellent sketch, although we are not aware that, upon these subjects, any additions are made to the knowledge of preceding writers.

The section on *polypi of the uterus* is an excellent practical lesson.

Dr. Gooch next gives a description of a disease with which the profession have hitherto been unacquainted, under the name of *irritable uterus*, "which (he says) is a painful and tender state of that organ, neither attended by nor tending to produce change in its structure; the causes (he adds) to which this disease has been attributed, and after the application of which it has occurred, are generally considerable bodily exertions at times when the uterus is in a susceptible state." For the treatment of this painful complaint, which is not uncommon, and which the author thinks bears a considerable analogy to the irritable mamma lately described by Sir Astley Cooper, he recommends bloodletting, rest, and narcotics, and more particularly henbane. In some cases he found opiate enemas, hipbaths, mercury, external irritation, and chalybeate waters, of advantage. This complaint is generally long and intractable.

The author next notices a species of *hemorrhage*, which occurs when the uterus is firmly contracted, and which depends on an excited state of the circulation. It is cured, or rather prevented, by depletion.

The volume concludes with an account of "the symptoms in children erroneously attributed to congestion of the brain;" and an appendix, containing a paper which was published in the Quarterly Review, entitled "Is the Plague contagious?" For this paper Dr. Gooch received the thanks of his Majesty's ministers.

In this work, the subject of the greatest importance which the author has discussed is undoubtedly that of peritoneal fever, both on account of the diversity of opinion as to the pathology of the disease, and because he has brought forward much matter towards its elucidation. We have no hesitation in pronouncing this to be a sound, elegant, and practically useful volume; and we strongly recommend those engaged in the responsible practice of obstetric medicine to make themselves well acquainted with its contents.

---

*An Account of the Morbid Appearances exhibited on Dissection in Disorders of the Trachea, Lungs, and Heart, with Pathological Observations, to which a Comparison of the Symptoms with the Morbid Changes has given rise.* By Thomas Mills, m.d. Honorary Fellow of the King and Queen's College of Physicians. —8vo. pp. 303. Cumming, Dublin, 1829.

The morbid anatomy of the viscera of the thorax is a subject which has of late years occupied, almost exclusively, the attention of some of the greatest pathologists whose names are recorded in the annals of medical science; and the works of Corvisart and Laennec, the labours of a lifetime, have embodied every thing known in reference to these diseases previous to their times, and have enriched and extended that knowledge, and rendered it so accurate and available, that it appears almost to border on temerity for any one, who has not enjoyed the most extended opportunities of observing these complaints, to attempt to add any thing new, or to overturn any of those opinions deduced from such a mass of experience; and the critic, who regards the title of a book like the present, is almost tempted to pass a condemnation on it, even before he has perused it. It ought, however, to be remembered that medicine has been greatly retarded in its progress towards improvement, by its professors being dazzled by the brilliancy of the opinions of men of great reputation, and not attempting to examine minutely the basis on which these rest. A book of cases, too, which is the nature of the work now before us, if they be narrated with truth, is always a valuable performance; for although separately they may be of little utility, yet, when combined and compared with others, they frequently lay the foundation for the most solid and useful improvements. The science of medicine, which is one in a very great degree of observation, must ever be indebted for its advancement to the continued exertions of many: "for thus it is that canals are formed and mountains levelled by the slender force of human beings."

The author is already known to the profession by a work on Disorders of the Brain, and in the present volume he has followed the same plan which he adopted in that, viz. of relating minutely many cases, and attempting, from the history of those which terminated unsuccessfully, in connexion with the morbid appearances on dissection, to explain their real nature, and to deduce the proper method of treatment: the latter he has also endeavoured still more to establish by the account of many whose successful course seems to support his views.

The work is divided into three portions, which comprehend the diseases of the trachea, lungs, and heart.

Under the head *Trachea* are classed two disorders, cynanche trachealis and cynanche maligna. The former he has repeatedly met with unaccompanied with that croupy noise in respiration by which it is commonly distinguished; and he asserts that, as far as his experience goes, there is no such thing as spasmodic croup, or croup unattended by inflammation; a statement with which many pathologists of the present day agree.* Of the acute form of croup, the author has related, with much accuracy of description, several interesting cases, one of which terminated fatally in twelve hours. The disease, in most of these instances, had been preceded by measles; and the author is of opinion that the inflammation spread by contiguity from the skin of the face to the mucous membrane of the trachea. Of the chronic form he has also given several examples.

On the nature of the disease, the author makes the following remarks:

"A spasm of the larynx or trachea, or of both, accompanies most cases of croup, and in many instances the danger is in proportion to its duration and the degree of its intensity. The spasms are induced by inflammation of the lining membrane of the windpipe, and their mildness or violence commonly depends on its degree and extent. To this general rule, however, there are

* It is true that the opinion that there is no such disease as spasmodic croup is not opposed even by CULLEN, the firm, if not pertinacious, supporter of the theory of spasm. For ourselves, we have no abstract hypothesis to contend for; but we have so often witnessed the sudden occurrence of the symptoms which are deemed most characteristic of croup, without any local or general signs of inflammatory action, and the equally sudden disappearance of such symptoms, without the intervention of any anti-inflammatory treatment, that we have no doubt whatever of the occasional existence of a pure form of spasmodic croup.—EDITORS.

exceptions; for, in one post-mortem examination at which I was present, the marks of inflammation were not unusually striking, yet the spasms were urgent, and apparently caused the death of the patient." (P. 22.)

His treatment of this dangerous and frequently fatal disorder corresponds to his views of its pathology.

"Bloodletting, general and topical, blistering, emetics, cathartics, and the hot bath, are the proper remedies, and they should be employed in quick succession. In the first instance, blood should be taken from the jugular vein or arm; leeches are then to be applied to the external fauces; an emeto-cathartic is to be immediately exhibited; and, as soon as possible, the patient is to be immersed for fifteen, thirty, or forty minutes in a hot bath, during which time the bleeding is to be encouraged from the orifices made by the leeches. If these remedies fail to produce relief, a blister is to be applied to the external fauces; or, what is more efficacious, boiling water, which often arrests the progress of the disease when employed at its onset. After depletion, calomel and opium should be given in large or small quantity, as may be deemed requisite." (P. 28.)

In chronic cases, the author found remarkable benefit produced by the use of the tartrite of antimony ointment, which he recommends in the strongest manner.

Of the numerous cases of disease of the *Lungs* which the author has related, it is rather difficult for us to give an idea, for they have not been classed with that scientific accuracy which their complicated and extended nature requires. They are, however, principally examples of inflammatory affections of the mucous membrane of the lungs, of ulceration, and the various changes produced in these organs by the disease denominated phthisis, complicated with inflammatory affections of the heart and other organs, and in one or two instances accompanied with cerebral effusion. He has also detailed several cases of tubercular phthisis, terminating in what he terms scirrhus and cancer of the lung; and has supported the theory that tubercles are nothing more or less than scrofulous lymphatic glands.

In cases of asthma, he found that ossification of the cartilages of the ribs, and chronic inflammation of the heart and of the lining membrane of the air-tubes, was a common occurrence; and, in some instances of obstruction of the lungs, he discovered, on dissection, a collection of watery fluid in the pericardium, without any disease of the heart itself.

A great many of the cases related are such as every practitioner must be in the daily habit of meeting with, and therefore it would be useless for us to make any extracts from them. It may be as well, however, to remark, that in those where the author found a difficulty in making a correct diagnosis, we think he might have been greatly assisted by the use of percussion and the stethoscope, and by an attention to those more minute symptoms which have been so precisely described by Laennec and Andral.

The author's opinions in reference to the origin of tubercles and the nature of phthisis, are best explained in his own words: "Were I allowed to form an opinion on this subject, grounded on observation and experience, I would say that the scrofulous tubercles of the lungs are lymphatic vessels, or a congeries of lymphatic vessels, called glands, in a state of inflammation and suppuration;" and the following are the grounds of this opinion:

"The existence of lymphatic glands in the lungs (the bronchial excepted,) has not been proved, but none deny the existence of lymphatic vessels. What are lymphatic glands but a congeries of lymphatic vessels joined together by cellular texture? In the bronchi and mesentery they are visible in a state of health; in the cervix, axillæ, groins, arms, &c. they are visible only in a state of disease, and then they are denominated scrofulous glands: so it is with the lungs; they too abound with lymphatic vessels and glands, but these are only apparent when enlarged by disease, and then they are designated scrofulous tubercles; and, whether in the lungs or the neck, they present the same symptoms and appearances." (P. 121.)

Of all the theories which have been advanced to account for the origin of tubercles, whether by Laennec and Andral, by Alison and Broussais, or by Jenner and Baron, this appears to be the most improbable, and is unsupported by any conclusive evidence whatsoever. The author, in proposing it, must have overlooked the numerous cases of Laennec, proving that tubercles may originate without any preceding inflammatory action. The assertion, also, of the author, that the lymphatic glands in the arms and axillæ are only visible in a state of disease, is certainly erroneous, for they can be readily detected by the hand of the anatomist.

Our author appears to have confounded old hepatisation and that condensation of the parenchyma of the lung which generally takes place in the immediate vicinity of a tuberculous excavation, for scirrhus and cancer. True scirrhus has been occasionally found in the bronchial glands, and more particularly in those patients who have died of open cancer of the mamma; but we are not aware of any case on record where it has been found to pervade the substance of the lung. The author's description is the following:

"In the left lung are numerous tubercles of different sizes, some of a cheesy or fatty nature, others in a state of ulceration; a considerable portion of this lung is converted into large irregular ulcers, the sides and edges of which are hardened and covered with purulent matter of a slate colour." (P. 93.)

On which he makes this comment:

"This is a case of tubercular phthisis, which terminated in extensive irregular ulceration and scirrhus of the left lung resembling cancer. Would not this and similar instances of scirrhus of the lungs tend to prove the existence of lymphatic glands in this organ?" (P. 94.)

The appearances ought, we think, to be attributed to that condition to which we have just alluded.

The cases of disease of the *Heart* are also numerous and interesting, but, like those of the lungs, they have not been classed under heads sufficiently definite and scientific either to enable the author to make proper deductions from them, or to render it easy for us to give an abstract of them. The greater portion are, however, instances of inflammation, acute or chronic, producing disorganization of the substance, and adhesions and other changes in the coverings of the heart, complicated, in some instances, with diseases of the valves; in others, with affections of the lungs, of the liver, and other viscera.

Two instances of angina pectoris are also recorded, in one of which the coronary arteries were completely ossified. The author has also given several well-marked examples of rheumatic inflammation of the heart.

The general method of treatment adopted in these cases may be gathered from the following paragraph:

"I have been emboldened to act with decision and firmness, unhesitatingly to employ evacuants where a weak and irregular pulse, and other symptoms of debility, have seemed to forbid their use; because dissection has taught me that, even where these symptoms existed, there was obstruction and inflammatory tendency, or actual inflammation, and that any other mode of treatment was either nugatory or destructive." (P. 267.)

From the cases related, the author thinks that he is entitled "to infer that diseases of the heart are often of an inflammatory character:" an inference, it may be well to remark, which is universally allowed at the present day; and we therefore think that he is hardly justified in stating that "they are usually classed with the affections called nervous, and considered as arising from debility: tonic and stimulating remedies, bark, wine, steel, valerian, &c. are usually administered."

The author also concludes, that angina pectoris generally proceeds from other affections besides ossification of the coronary arteries; a deduction which is by no means an original one; for Dr. HAYGARTH, long previous to his time, attributed it to inflammation of the mediastinum; Dr. HOOPER, to diseases of the pericardium; and Dr. HOSACK and Dr. FORBES to general plethora. Some have considered it to be a neuralgic affection of the cardiac nerves; and even Dr. PARRY, who first described the disease as depending on ossification of the coronary arteries, admits that there is also present an accumulation of blood in the heart and its large vessels.

This work, of which we have endeavoured to give our readers the outline, is certainly greatly deficient in arrangement; for, after the minute divisions which previous pathologists have established in reference to these complicated disorders, we should hardly have expected to find any author arranging them under two such general heads as "diseases of the lungs" and "diseases of the heart;" nor, as we have already stated, do we think it possible to deduce from so many dissimilar affections, mingled together in this way, those practical inferences which might otherwise have been drawn from them.

The volume is, however, valuable on account of the accuracy with which the cases are detailed, and more particularly for the description of the morbid appearances on dissection. The treatment of the patients, too, appears to us to have been highly judicious; and, on these accounts, we think that the author is well entitled to the attention of pathologists and practitioners.

